# Three-Dimensional Reconstruction of Coronary Arteries and Its Application in Localization of Coronary Artery Segments Corresponding to Myocardial Segments Identified by Transthoracic Echocardiography

**DOI:** 10.1155/2013/783939

**Published:** 2013-11-20

**Authors:** Chunyan Zhong, Yanli Guo, Haiyun Huang, Liwen Tan, Yi Wu, Wenting Wang

**Affiliations:** ^1^Department of Ultrasound, Southwest Hospital, Third Military Medical University, Chongqing, China; ^2^School of Basic Medicine, Third Military Medical University, Chongqing, China

## Abstract

*Objectives.* To establish 3D models of coronary arteries (CA) and study their application in localization of CA segments identified by Transthoracic Echocardiography (TTE). *Methods.* Sectional images of the heart collected from the first CVH dataset and contrast CT data were used to establish 3D models of the CA. Virtual dissection was performed on the 3D models to simulate the conventional sections of TTE. Then, we used 2D ultrasound, speckle tracking imaging (STI), and 2D ultrasound plus 3D CA models to diagnose 170 patients and compare the results to coronary angiography (CAG). *Results.* 3D models of CA distinctly displayed both 3D structure and 2D sections of CA. This simulated TTE imaging in any plane and showed the CA segments that corresponded to 17 myocardial segments identified by TTE. The localization accuracy showed a significant difference between 2D ultrasound and 2D ultrasound plus 3D CA model in the severe stenosis group (*P* < 0.05) and in the mild-to-moderate stenosis group (*P* < 0.05). *Conclusions.* These innovative modeling techniques help clinicians identify the CA segments that correspond to myocardial segments typically shown in TTE sectional images, thereby increasing the accuracy of the TTE-based diagnosis of CHD.

## 1. Introduction

The incidence of coronary heart disease (CHD) has increased in China. Due to technical advances, transthoracic echocardiography (TTE) and new TTE-based techniques (such as myocardial contrast echocardiography and myocardial quantitative analysis) have become important diagnostic and prognostic modalities in CHD [1]. In contrast to CAG, TTE indicates myocardial ischemia by identifying abnormal segmental movements of the ventricular wall in CHD patients and cannot directly display the occluded coronary arteries. Therefore, the use of TTE is limited in the diagnosis and assessment of CHD [2, 3]. In addition, TTE has difficulty in displaying the blood flow in the coronary arteries due to the complicated paths, narrow lumina, and frequent anatomic variations of the coronary arteries. In the present study, 3D visible models of human coronary arteries were established based on the Chinese visible human (CVH) datasets and coronary CT images. These models provide 2D and 3D anatomical bases for the localization of coronary artery segments that correspond to the 17 myocardial segments identified by conventional TTE. This work may help directly identify occluded coronary arteries by using TTE to display myocardial segmental abnormalities in CHD patients.

## 2. Materials and Methods

### 2.1. Acquisition of Heart Slice Images

The CVH datasets comprised 286 successive sections of the heart from the first female CVH. The slice thickness was 0.5 mm, and the image resolution was 3072 × 2048 pixels. The CT datasets comprised 266 CT images of the heart obtained during CAG. The subjects included 3 men and 2 women without any heart disease, who were aged 25–48 years and had a medium body height. The slice thickness was 0.5 mm, and the image resolution was 512 × 512 pixels.

### 2.2. 3D Reconstructions of the Coronary Arteries

#### 2.2.1. Data Partitioning

The “magnetic lasso” and the “polygonal lasso” in Photoshop CS2 9.0 were used to permit data partitioning of the coronary artery imaging data in the CVH datasets. Then, different gray values were assigned to different branches of the coronary arteries. These images were saved in PNG format.

Partitioning tools such as “threshold segmentation,” “magnetic lasso,” and “semiautomatic identification” in the Amira 5.2.1 software were used to segment the coronary arteries in duel source computed tomography angiography (DSCTA). Then, different branches of the coronary arteries were labeled. The labels were colored with different RGB values and saved in AM format.

#### 2.2.2. Reconstruction

The hardware used in our research included the following equipment: a dual-core CPU with AMD Hyper Transport TM technology, a 64-bit Windows XP operating system, a Dual Graphic Quadro FX 4500 graphics card, 64 MB of video memory, support for OpenGL1.4, 16.0 GB of physical memory, and 4.0 GB of virtual memory. We also used Amira 5.2.1 software.

A total of 286 layers of CVH partitioning data were imported into the Amira 5.2.1 software, and the “Channel 1” mode was selected as the gray value channel. The selected voxel parameter values were 0.167/0.167/1.0 in the *X*/*Y*/*Z* directions, respectively, and surface rendering was started by the software. Color layers were imported into the “color field,” and volume rendering was completed. Surface rendering and volume rendering were obtained from the same data and used the same coordinates, allowing the software to match them together without errors. The “am” DSCTA datasets were imported into Amira 5.2.1. We then clicked the “File” button to choose the correct “am” file, which was renamed, and completed the surface rendering. All the “am” files were selected and opened in Amira 5.2.1, and volume rendering was then completed.

The software could reconstruct 3D models automatically by right clicking on the surface rendering mode, choosing “Labeling—Label field,” and clicking the “SurfaceGen” button. Then, “SurfaceView” was clicked to display the models. Clicking the “OrthoSlice” button displayed three planes in the *xy*/*xz*/*yz* directions. “Display—ObliqueSlice” was chosen to control the virtual spin ball and to choose any desired angle and slice.

### 2.3. Establishment of 3D Visible Models of the Coronary Arteries

Coronary artery images from the surface and volume renderings were displayed in combination to establish the 3D models of the coronary arteries. According to the myocardial segmentation method stipulated by the American Heart Association, virtual dissection was performed using Amira 5.2.1 on the 3D visible models to simulate conventional TTE sections, including a long-axis view of the left ventricle, a 4-chamber apical view, a 2-chamber apical view, and a short-axis view of the left ventricle (including a short-axis view of the basal segment, a short-axis view of the left ventricular papillary muscle, a short-axis view of the apex, and a top view of the apex).

### 2.4. Acquisition of Normal Ultrasound Images

Fifteen healthy medical students underwent TEE using a Philips IE33 color Doppler scanner with an S5-1 heart probe to obtain conventional sectional images including a long-axis view of the left ventricle, a four-chamber apical view, a two-chamber apical view, and a short-axis view of the left ventricle (including a short-axis view of the basal segment, a short-axis view of the left ventricular papillary muscle, a short-axis view of the apex, and a top view of the apex). All images were stored in AVI or DICOM format on compact discs (CDs).

### 2.5. Clinical Application

#### 2.5.1. Studying Objects

A total of 170 patients (95 males and 75 females), aged between 40 and 82 years (61.2 ± 8.9), with a positive history or clinical suspicion of CHD, were treated at the Southwest Hospital between January 2011 and October 2011.

#### 2.5.2. 2D Ultrasound Images

Ultrasound images were acquired according to the methods described in Section 2.4. Coronary lesions were diagnosed by the sonographic identification of abnormal segmental ventricular wall motion. The examiners were unaware that ultrasonography was being performed for the purposes of this study.

#### 2.5.3. Location of Coronary Artery Lesions Using STI

After image acquisition, CMQ in the QLab software was used to open a 4-chamber apical view in diastole. In these images, 3 points (the anterior mitral valve annulus, the posterior mitral valve annulus, and the apex cordis) were selected and then divided into 7 segments (segments 3, 6, 9, 12, 14, 16, and 17) automatically using the AP4 mode. The shape and direction of these areas of interest were adjusted to conform to the myocardial thickness. The same methods were used with AP2 mode in a 2-chamber apical view to identify 7 segments (segments 1, 4, 7, 10, 13, 15, and 17). The same methods were then used with the AP3/LAX mode in the long-axis view of the left ventricle to view segments 2, 5, 8, 11, 14, 16, and 17.

#### 2.5.4. Locating Coronary Artery Lesions Using 3D Models of the Coronary Arteries

For slices with echocardiographic evidence of abnormal segmental ventricular wall motion, “ObliqueSlice” was clicked in the volume rendering of the coronary arteries to simulate different ultrasound image angles by rotating the spin ball in the software. For each patient, an average of 25 minutes was needed to analyze the lesioned coronary artery and its branches.

#### 2.5.5. CAG Examination

The CAG examination was performed to confirm the results of the ultrasound and 3D coronary artery models. The Judkin method was used for the CAG examination. When >50% stenosis was present, the CAG examination was considered positive; otherwise, it was considered negative.

#### 2.5.6. Statistical Analysis

The diagnostic accuracy rates were compared between the following: 2D ultrasound and 2D ultrasound plus 3D coronary artery models and STI and 2D ultrasound plus 3D coronary artery models. The data were analyzed using SPSS 13.0 software. Numerical data were compared using the *χ* ^2^ test. A *P* value of <0.05 was deemed statistically significant.

## 3. Results

**Figure 1:**
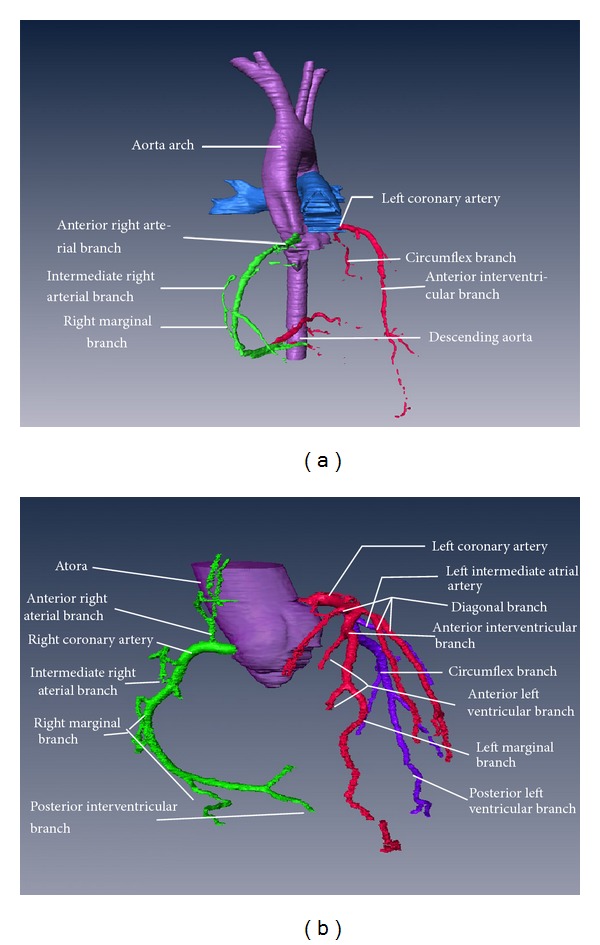
3D visible models of the coronary arteries based on the CVH and cardiac CT datasets. (a) Coronary artery model based on the CVH dataset. (b) Coronary artery model based on the cardiac CT dataset.

**Figure 2:**
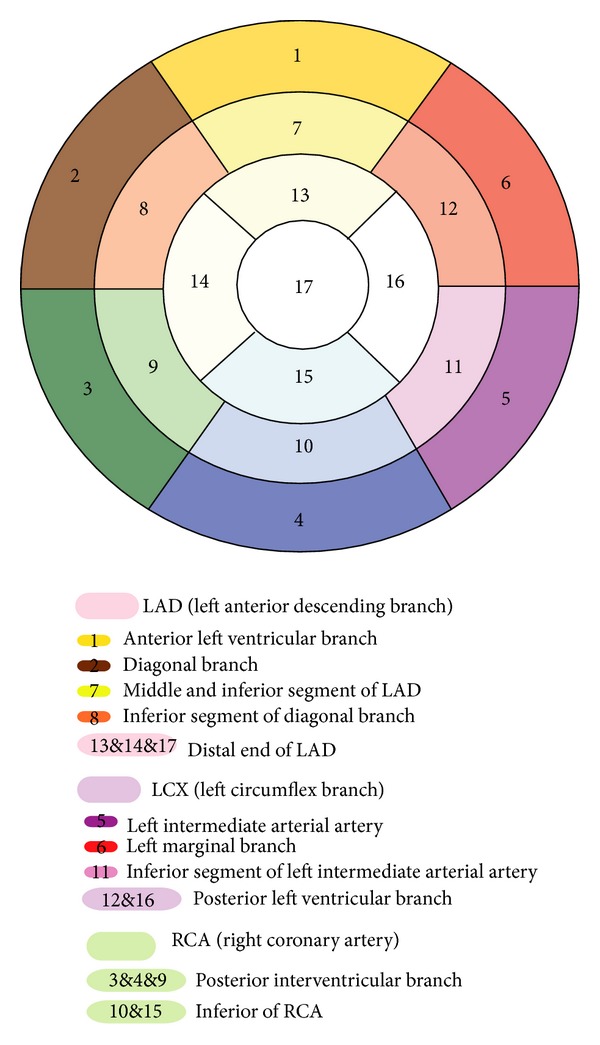
Our identification of the coronary artery segments corresponding to the 17 myocardial segments.

In the present study, 3D visible models of the human coronary arteries were established on the basis of the CVH and clinical CT image datasets (Figures 1(a) and 1(b)). These models were right dominant, which is representative of the coronary artery anatomy in approximately 2/3 of Chinese people. These models can distinctly display the trunk and main branches of the normal human coronary arteries. The CT-based coronary artery model can display most of the tertiary branches of the coronary arteries. Moreover, these models combined the 3D structure of the coronary arteries and their branches with gross anatomic and 2D sections of the heart to simulate conventional TTE sections. In these models, the coronary artery segments corresponding to the 17 myocardial segments identified by conventional TTE can be displayed distinctly and recognized accurately (Figure 2).

### 3.1. Dissection of the Parasternal Short-Axis View of the Left Ventricle on 3D Visible Models of the Coronary Arteries and Comparison with TTE

The parasternal short-axis view of the left ventricle usually displayed blood supply to the left ventricular wall, helping to identify the site of myocardial ischemia and infarction. Various parts of the left ventricular wall could be displayed along the short axis. At the mitral leaflet level, the left ventricular wall near the cardiac base was displayed, and the middle and apical parts of the left ventricular wall were displayed at the papillary muscle and apical levels, respectively.

#### 3.1.1. Parasternal Short-Axis View of the Left Ventricle at the Level of the Mitral Leaflet Segments 1–6 of the Left Ventricular Myocardium Was Displayed

**Figure 3:**
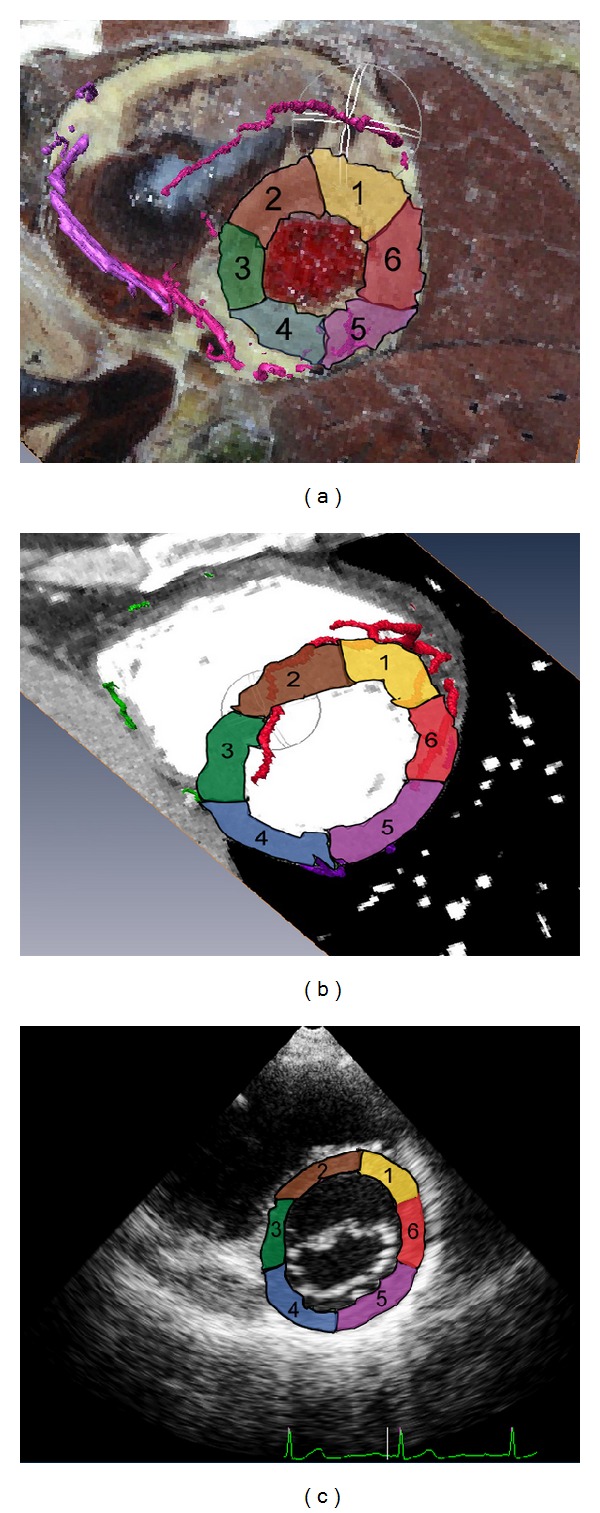
Short-axis sections of left ventricle at the level of the mitral leaflet. (a) Fusion display of the CVH dataset and the coronary artery model. (b) Fusion display of the cardiac CT dataset and the coronary artery model. (c) Short-axis section of the left ventricle at the level of the mitral leaflet revealed by echocardiography.

On the CVH-based coronary artery model, the upper and middle segments of the anterior descending branch of the left coronary artery supplied blood to segments 1 and 2 (i.e., the anterior septal base and the anterior wall base, resp.), the middle and inferior segments of the left circumflex branch supplied blood to segments 3 and 4 (i.e., the base of the lateral wall and the base of the posterolateral wall, resp.), and the right coronary artery supplied blood to segments 5 and 6 (i.e., the base of the posteroinferior/inferior wall). The CT-based coronary artery model showed that the anterior left ventricular branch supplied blood to segment 1, the diagonal branch supplied blood to segment 2, the posterior interventricular branch supplied blood to segments 3 and 4, the left intermediate artery supplied blood to segment 5, and the left marginal branch supplied blood to segments 5 and 6 (Figures 3(a) and 3(b)).

#### 3.1.2. Parasternal Short-Axis View of the Left Ventricle at the Level of the Papillary Muscle

**Figure 4:**
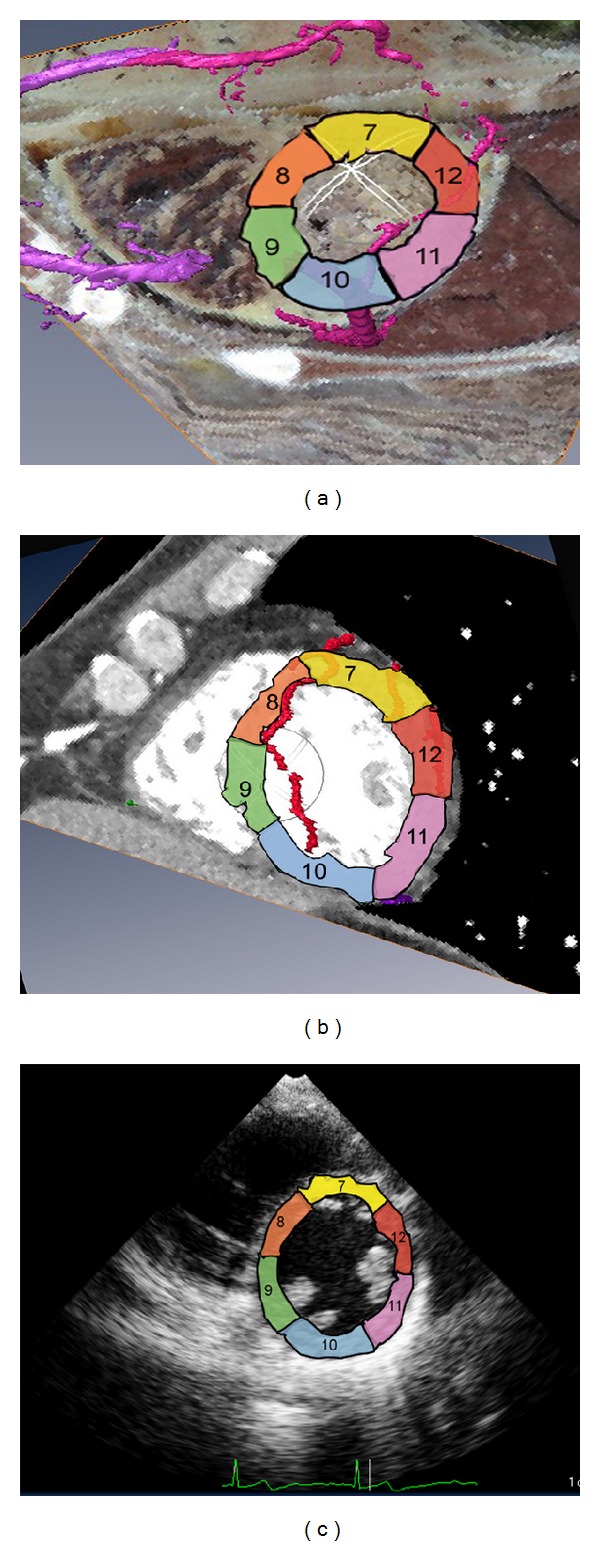
Short-axis sections of the left ventricle at the level of the papillary muscle. (a) Fusion display of the CVH dataset and the coronary artery model. (b) Fusion display of the cardiac CT dataset and the coronary artery model. (c) Short-axis section of the left ventricle at the level of the papillary muscle revealed by echocardiography.

Myocardial segments 7–12 of the left ventricle were displayed, including the posterior septal base, the septal base, the anterior septum, the anterior wall, the lateral wall, and the posterolateral wall. On the CVH-based coronary artery model, the middle and inferior segments of the anterior descending branch of the left coronary artery supplied blood to segments 7 and 8, respectively, the left circumflex branch supplied blood to segments 9 and 10, and no vessels penetrated segments 11 and 12. On the CT-based coronary artery model, the middle and inferior segments of the anterior descending branch trunk supplied blood to segment 7, the interior segment of the diagonal branch supplied blood to segment 8, the posterior interventricular branch supplied blood to segment 9, the inferior right coronary artery supplied blood to segment 10, the inferior segment of the left intermediate atrial artery supplied blood to segment 11, and the posterior left ventricular branch supplied blood to segment 12 (Figures 4(a) and 4(b)).

#### 3.1.3. Parasternal Short-Axis View of the Left Ventricle at the Level of the Apex

**Figure 5:**
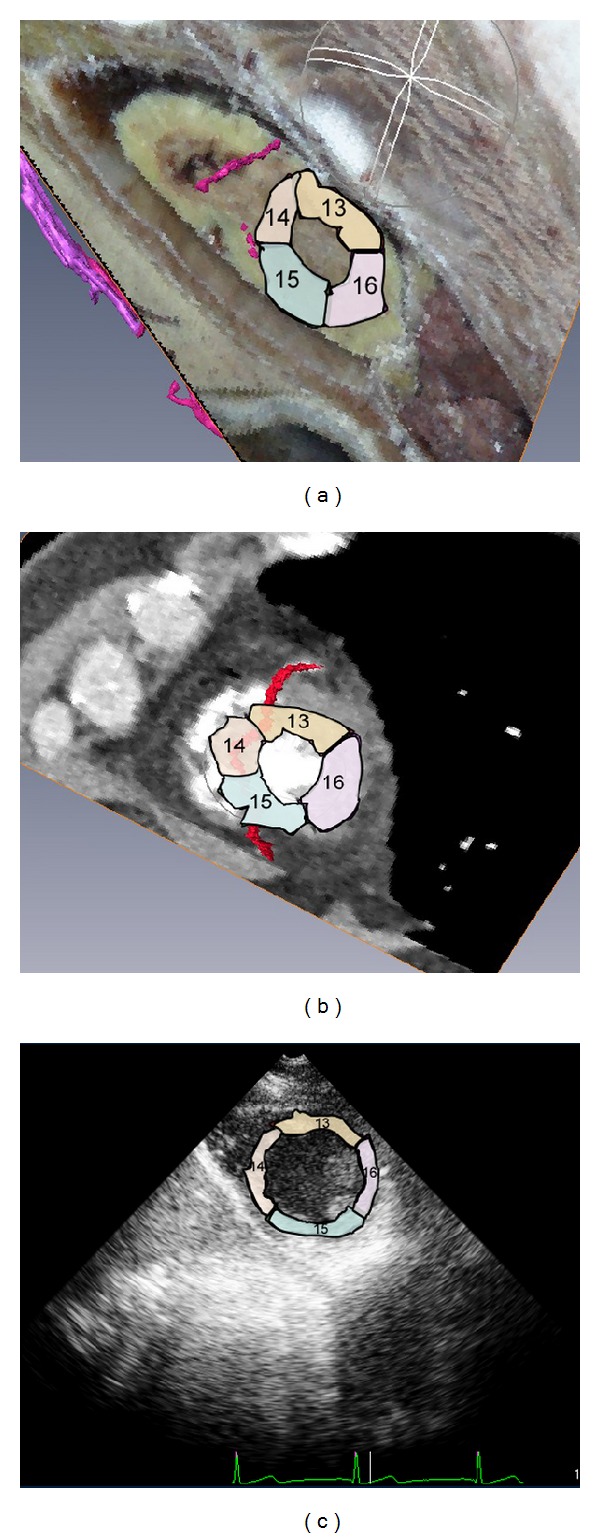
Short-axis sections of the left ventricular apex. (a) Fusion display of the CVH dataset and the coronary artery model. (b) Fusion display of the cardiac CT dataset and the coronary artery model. (c) Short-axis section of the left ventricular apex revealed by echocardiography.

Myocardial segments 13–17 of the left ventricle were displayed, including the posteroinferior wall, the inferior wall, the posterior septum, the septum, and the apex of the anterior wall. Coronary artery models based on the CVH and CT datasets showed that the distal end of the left anterior descending branch supplied blood to segments 13, 14, and 17, the inferior right coronary artery supplied blood to segment 15, and the posterior left ventricular branch supplied blood to segment 16 (Figures 5(a) and 5(b)).

### 3.2. Visualization of the Heart in a 4-Chamber Apical View Using the Coronary Artery Model and Comparison with TTE Studies

**Figure 6:**
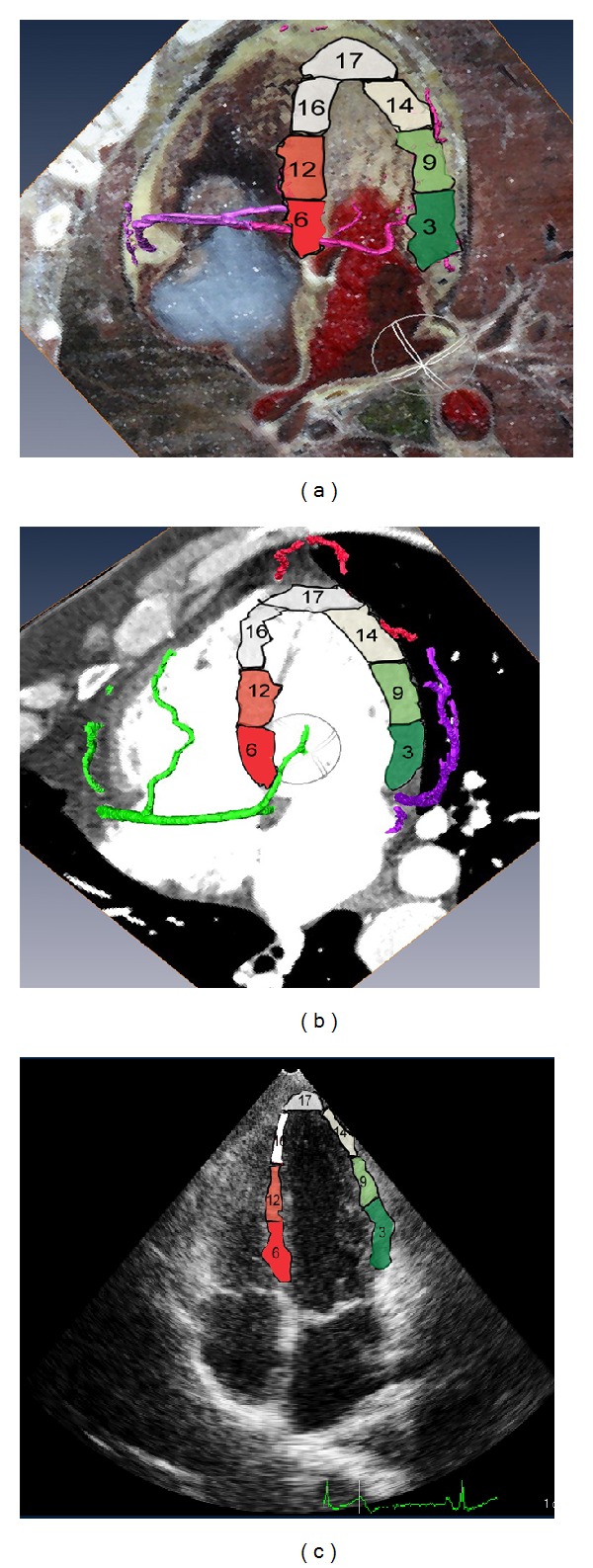
Four-chamber heart sections. (a) Fusion display of the CVH dataset and the coronary artery model. (b) Fusion display of the cardiac CT dataset and the coronary artery model. (c) Two-chamber heart section revealed by echocardiography.

Myocardial segments 3, 9, 13, 16, and 17 of the left ventricle were displayed in the 4-chamber apical view. This view usually displays the posterior septum and clearly displays the morphology and motion of the anterolateral wall of the left ventricle. On the coronary artery models based on the CVH and CT datasets, the posterior interventricular branch supplied blood to segments 3 and 9, the distal end of left anterior descending branch supplied blood to segments 13 and 17, and the posterior left ventricular branch supplied blood to segment 16 (Figures 6(a) and 6(b)).

### 3.3. Localization Accuracy of the 3 Methods for Lesions in the Coronary Arteries

The CAG showed that there were 141 patients with coronary stenosis and 29 patients without stenosis. In these 141 patients, 43 patients were classified as having severe stenosis, with at least 75% blockage of 1 coronary artery. This group contained 65 males and 14 females. The other 97 patients were classified as having mild-to-moderate stenosis. This group contained 65 males and 33 females, who ranged in age from 40 to 82 years. The results of all 4 diagnostic methods in each group are shown in Tables 1 and 2. The localization accuracy of the 2D ultrasound, STI and, the 2D ultrasound plus 3D coronary artery model was 69.8% (30/43), 88.4% (38/43), and 88.4% (38/43), respectively, in the severe stenosis group (Table 3). The localization accuracy in the mild-to-moderate stenosis group was 66.3% (65/98) for 2D ultrasound, 90.8% (89/98) for STI, and 89.8% (88/98) for the 2D ultrasound plus 3D coronary artery model (Table 4). The results showed a significant difference between the 2D ultrasound and 2D ultrasound plus 3D coronary artery model in the severe stenosis group (*P* < 0.05) and in the mild-to-moderate stenosis group (*P* < 0.05). Additionally, there was no significant difference between the STI and 2D ultrasound plus 3D coronary artery model.

## 4. Discussion

CHD is a common cardiovascular disease and the leading cause of death in western countries. The incidence of CHD has tended to increase in China. Therefore, the early diagnosis and posttreatment assessment of CHD are clinically significant. TTE provides important diagnostic and prognostic information for CHD because of its lack of invasiveness, simple examination technique, and ability to provide ample anatomical and hemodynamic information. Abnormal segmental ventricular wall motion is a sensitive and specific indicator for myocardial ischemia. To facilitate data exchange among various imaging modalities, including ultrasonography, radionuclide imaging, MRI, and CT, the American Heart Association (AHA) has suggested a myocardial segmentation method that divides the myocardium into 17 segments [4]. In contrast to other imaging modalities, TTE suggests the presence of an occluded coronary artery by detecting the abnormal motion of the ventricular wall. The identification of the coronary artery segments that correspond to the myocardial segments directly on TTE 2D sections is difficult. Therefore, 3D visible models of the human coronary arteries were established in the present study on the basis of the CVH dataset and a normal cardiac CT dataset, thereby providing both 2D and 3D anatomic bases for localizing the coronary artery segments corresponding to the 17 myocardial segments identified by conventional TTE.

### 4.1. Establishment of 3D Visible Models of the Coronary Arteries

The coronary arteries are the first pair of branches originating from the ascending aorta and provide the primary supply of blood to the heart. The trunk of the left coronary artery originates from the left coronary sinus. The left coronary artery passes between the pulmonary artery and left auricle and bifurcates into the anterior descending branch and the left circumflex branch. The anterior descending branch divides into the left arterial cone, the right ventricular anterior branch, the left ventricular anterior branch, the anterior septal artery, and the diagonal branch. The left circumflex branch divides into the left ventricular anterior branch, the marginal branch, the left atrial branch, and the left ventricular posterior branch. The right coronary artery originates from the anterior aortic sinus; its branches include the right arterial cone, the right ventricular anterior branch, the right marginal branch, the right ventricular posterior branch, the right atrial branch, the atrioventricular nodal artery, the posterior descending branch, and the left atrial posterior branch. Three-dimensional visualizations of the coronary arteries are challenging because of the large number of thin branches from these vessels, which have a highly variable anatomy. The CVH has been demonstrated to help individuals master medical imaging technologies. Based on the CVH, virtual human organs and tissues with real anatomical information can be modeled, thereby facilitating 3D visualizations of human anatomy [5–7]. In the present study, we used the cardiac dataset from the first CVH reported by the Computerized Medicine Laboratory of the Third Military Medical University in February 2003 [8, 9]. The 3D visible model of the coronary arteries that was based on the CVH dataset clearly displayed the 3D structure of the left and right coronary arteries and their major branches, such as the anterior interventricular branch, left circumflex branch, arterial cone, and posterior interventricular branch. Because the CVH dataset contains delicate anatomical information in 2D sections, the courses and locations of the coronary arteries can be displayed distinctly on the 3D visible model using software-based dissection, suggesting that the CVH dataset can provide high-quality, well-aligned 2D data for establishing 3D visible models of the human coronary arteries.

Due to the highly variable anatomy of the coronary arteries, a single CVH dataset is not adequate for a 3D visualization study of the coronary arteries. Computed tomography angiography (CTA) of the coronary arteries has been shown to clearly display the trunk and branches of the coronary arteries, particularly the trunk of the main coronary artery, the proximal segment of the right coronary artery, the proximal segment of the anterior descending branch, and the proximal segment of the left circumflex branch [10, 11]. CTA is less effective than CAG in displaying the distal segment of the right coronary artery, the distal segment of the left anterior descending branch, and the distal segment of the circumflex branch. The CTA dataset complements the CVH dataset to display the 3D structure of the coronary arteries. Based on CTA datasets from 5 subjects without heart disease, 5 3D visible models of the coronary arteries were established. Compared with the 3D visible model based on the CVH dataset, the models based on the CTA datasets displayed the coronary arteries and their branches clearly, including the trunk and branches of the left and right coronary arteries. The CTA-based models combined with the CT-based 2D sectional images complement the 3D model that was based on the CVH dataset, showing the delicate course of the coronary artery branches and providing a basis for fusing and converting cardiac CT and echocardiographic images to 3D models.

### 4.2. 3D Visible Models of the Coronary Arteries Provide 2D and 3D Anatomical Bases for the TTE-Based Diagnosis of CHD

Recently, virtual reality medicine (VRM) has been widely used in medicine [12]. Using VRM, anatomical structures can be viewed with 3D reconstructions in any direction, and virtual dissections can be performed as needed to visualize intracardiac structures and vessels. Therefore, VRM can provide 2D and 3D anatomical bases for various imaging modalities, such as ultrasonography, CT, and MRI [13, 14]. In the present study, 3D visual models of the coronary arterial anatomy were established on the basis of the CVH and cardiac CT imaging datasets. These models can clearly display the 3D courses and spatial relationships of the coronary arteries and can be dissected and rotated in any direction with the use of 3D reconstruction software. We further simulated conventional TTE imaging of the left ventricle in various views to display the coronary arteries with their branches corresponding to the 17 segments of the left ventricle and compared these results with conventional TTE sectional images. To the best of our knowledge, this report is the first 3D visualization study of the coronary arteries that was based on CVH and CT imaging datasets and a comparison with TTE. In our research, combining 2D ultrasound and 3D coronary artery models can significantly improve the accuracy with which lesioned coronary arteries are located. The accuracy of 2D ultrasound plus the 3D coronary artery model was similar to that of STI. Thus, we can include these models in a diasonograph to display the coronary arteries in 2D ultrasound images without image postprocessing. This technique could permit the diagnosis of coronary artery disease using 2D ultrasound imaging.

In summary, 3D visible models of the coronary arteries can simulate conventional TTE imaging. These models can clearly display the coronary artery branches corresponding to the 17 segments of the left ventricle, thereby providing anatomical landmarks for 2D echocardiography and helping clinicians identify coronary artery segments through myocardial segments displayed by TTE.

## Figures and Tables

**Table 1:** Number of lesioned coronary artery branches detected by the 4 imaging methods (severe stenosis group).

Method	LAD	LCX	RCA	Multivessel lesion	Total
2D ultrasound	6	2	3	32	43
STI	7	3	5	28	43
2D ultrasound plus 3D model	9	4	5	25	43
CAG	9	4	6	24	43

LAD: left anterior descending branch; LCX: left circumflex branch; RCA: right coronary artery; CAG: coronary angiography; STI: speckle tracking imaging.

**Table 2:** Number of lesioned coronary artery branches detected by the 4 imaging methods (light-moderate stenosis group).

Method	LAD	LCX	RCA	Multivessel lesion	Total
2D ultrasound	20	5	11	62	98
STI	21	7	11	61	98
2D ultrasound plus 3D model	23	6	11	58	98
CAG	24	6	11	57	98

LAD: left anterior descending branch; LCX: left circumflex branch; RCA: right coronary artery; CAG: coronary angiography; STI: speckle tracking imaging.

**Table 3:** Accuracy of localization for lesioned coronary arteries using 3 different imaging methods (severe stenosis group).

Methods	CAG results		Accuracy of localization (%)
	True	False	
2D ultrasound	30	13	69.8*
STI	38	5	88.4
2D ultrasound plus 3D model	38	5	88.4

^∗^ *P*  <  0.05 for 2D ultrasound versus 2D ultrasound plus 3D model.

**Table 4:** Accuracy of localization for lesioned coronary arteries using 3 different imaging methods (severe stenosis group).

Methods	CAG results		Accuracy of localization (%)
	True	False	
2D ultrasound	65	33	66.3*
STI	89	9	90.8
2D ultrasound plus 3D model	88	10	89.8

^∗^ *P*  <  0.05 2D ultrasound versus 2D ultrasound plus 3D model.
